# The capsule of *Bacillus anthracis* protects it from the bactericidal activity of human defensins and other cationic antimicrobial peptides

**DOI:** 10.1371/journal.ppat.1010851

**Published:** 2022-09-29

**Authors:** David K. O’Brien, Wilson J. Ribot, Donald J. Chabot, Angelo Scorpio, Steven A. Tobery, Tanya M. Jelacic, Zhibin Wu, Arthur M. Friedlander

**Affiliations:** 1 United States Army Medical Research Institute of Infectious Diseases, Frederick, Maryland, United States of America; 2 Institute of Human Virology, University of Maryland Biotechnology Institute, Baltimore, Maryland, United States of America; 3 Department of Medicine, Uniformed University of Health Services, Bethesda, Maryland, United States of America; University of Illinois, UNITED STATES

## Abstract

During infection, *Bacillus anthracis* bacilli encounter potent antimicrobial peptides (AMPs) such as defensins. We examined the role that *B*. *anthracis* capsule plays in protecting bacilli from defensins and other cationic AMPs by comparing their effects on a fully virulent encapsulated wild type (WT) strain and an isogenic capsule-deficient *capA* mutant strain. We identified several human defensins and non-human AMPs that were capable of killing *B*. *anthracis*. The human alpha defensins 1–6 (HNP-1-4, HD-5-6), the human beta defensins 1–4 (HBD-1-4), and the non-human AMPs, protegrin, gramicidin D, polymyxin B, nisin, and melittin were all capable of killing both encapsulated WT and non-encapsulated *capA* mutant *B*. *anthracis*. However, non-encapsulated *capA* mutant bacilli were significantly more susceptible than encapsulated WT bacilli to killing by nearly all of the AMPs tested. We demonstrated that purified capsule bound HBD-2, HBD-3, and HNP-1 in an electrophoretic mobility shift assay. Furthermore, we determined that the capsule layer enveloping WT bacilli bound and trapped HBD-3, substantially reducing the amount reaching the cell wall. To assess whether released capsule might also play a protective role, we pre-incubated HBD-2, HBD-3, or HNP-1 with purified capsule before their addition to non-encapsulated *capA* mutant bacilli. We found that free capsule completely rescued the *capA* mutant bacilli from killing by HBD-2 and -3 while killing by HNP-1 was reduced to the level observed with WT bacilli. Together, these results suggest an immune evasion mechanism by which the capsule, both that enveloping the bacilli and released fragments, contributes to virulence by binding to and inhibiting the antimicrobial activity of cationic AMPs.

## Author summary

*Bacillus anthracis* causes anthrax after spores infect the skin, respiratory tract, or gastrointestinal tract. Antimicrobial peptides (AMPs), such as defensins, are a first line of host defense that *B*. *anthracis* encounters in all of these tissues. *B*. *anthracis* bacteria are covered by a capsule that protects them from being engulfed and destroyed by phagocytic immune cells. In this study, we found that the capsule also provides protection from AMPs. An encapsulated *B*. *anthracis* strain is resistant to killing by multiple AMPs from humans and other species compared to an otherwise identical strain that is not encapsulated. By binding defensins the capsule surrounding the bacilli reduces the amount that gets to the bacterial cell wall where it can do damage. *B*. *anthracis* bacteria release large fragments of capsule in the host during infection and during growth in culture. We found that purified released capsule can bind defensins and reduce killing of non-encapsulated *B*. *anthracis*. Thus, both capsule covering the bacteria and capsule shed by the bacteria can contribute to the pathogenicity of *B*. *anthracis* by providing protection from AMPs. Our study reveals a new mechanism by which *B*. *anthracis* capsule contributes to virulence.

## Introduction

*Bacillus anthracis* is the causative agent of cutaneous, gastrointestinal (GI), and inhalational anthrax [1]. Cutaneous anthrax occurs when spores enter through breaks in the skin. GI anthrax occurs after the ingestion of contaminated meat. Inhalational anthrax occurs after spores are inhaled into the lungs and trafficked to lymph nodes where they are thought to germinate into bacilli and begin expressing virulence factors. The primary virulence factors of *B*. *anthracis* are its toxins encoded on plasmid pXO1 [2] and its capsule encoded on plasmid pXO2 [3,4]. *B*. *anthracis* capsule is an anionic polymer of γ-linked D glutamic acid residues. Capsule is both covalently linked to the peptidoglycan of the cell wall and released from it by the enzyme capsule depolymerase (CapD) [5–8]. Capsule provides a multifaceted defense against the host immune system. Encapsulation protects the bacilli from phagocytosis by immune cells [9–11] and inhibits dendritic cell maturation by shielding more pro-inflammatory components on the bacillus surface [12]. Purified released capsule has been shown to restore the virulence of an attenuated CapD mutant strain in mice [5]. Purified free capsule has also been shown to have inhibitory effects on dendritic cells [13]. In this study we investigate the effects of capsule on another branch of host defense, antimicrobial peptides (AMPs).

*B*. *anthracis* encounters AMPs during infection by all three routes since AMPs are produced by epithelial cells in the skin, respiratory tract, and GI tract and by neutrophils and natural killer cells [14]. Indeed, an older study identified a basic polypeptide extracted from mammalian tissues and neutrophils that had both *in vitro* antimicrobial activity and *in vivo* efficacy against anthrax infection in mice [15]. AMPs are broad-spectrum antimicrobials active against both Gram-positive and -negative bacteria, fungi, and certain viruses [16,17] that may also act as signaling molecules for the innate and adaptive immune systems [18,19]. They are produced by microbes, arthropods, amphibians, mammals, and plants and exert their bactericidal activity in a variety of ways including disruption of bacterial membranes, formation of pores in the membrane, and by damage to intracellular targets [17,20]. Humans produce three types of cationic AMPs: defensins, the cathelicidin LL-37, and histatins, histidine rich peptides that are found exclusively in saliva [21]. Human defensins are characterized by three intramolecular disulfide bonds forming a triple-stranded beta sheet and are divided into two groups, alpha and beta [22,23]. There are six known human alpha defensins (HNP-1-4 and HD-5-6) [24] and more than thirty human beta defensins [25], four of which (HBD-1-4) have been extensively studied [26–29]. HNP-1-4 are found in the azurophilic granules of neutrophils [30], while HD-5-6 are found mainly in the granules of Paneth cells of the small intestine [31,32]. The human beta defensins are expressed mainly in epithelial tissues and have been reported in the lungs, pancreas, kidney, skin, tonsils, leukocytes, and testes [27,33–36]. Previous work has indicated that *B*. *anthracis* can be sensitive to human defensins. HBD-3, but not HBD-1 or –2, has been reported to be bactericidal for the non-encapsulated Sterne strain of *B*. *anthracis* [37], while in another report, modest bactericidal activity against the Sterne strain has been noted for HBD-2 and HNP-2 [38]. HNP-1 and HNP-2 have also been shown to have activity against the non-encapsulated Sterne strain [39] and alpha defensins have been linked to killing of both encapsulated and non-encapsulated *B*. *anthracis* strains by human neutrophils [40]. HBD-2 and -3 have also been demonstrated to have bactericidal activity against various *Bacillus* species other than *B*. *anthracis* [41]. In this study, we assess and compare the bactericidal effects of human alpha and beta defensins and various non-human AMPs against the fully virulent wild type (WT) encapsulated *B*. *anthracis* Ames strain and an isogenic non-encapsulated *capA* mutant strain.

While defensins and other cationic AMPs are highly bactericidal, many pathogenic bacteria have evolved ways to circumvent them [42,43]. In this report, we present evidence that *B*. *anthracis* capsule confers resistance to many human defensins and some non-human AMPs. We show that a WT encapsulated *B*. *anthracis* strain is more resistant to killing than an isogenic non-encapsulated strain. We demonstrate that purified capsule binds HBD-2, HBD-3, and HNP-1 *in vitro*. Further, we demonstrate that while HBD-3 binds to the membrane of both encapsulated and non-encapsulated bacilli, the capsule layer surrounding encapsulated bacilli prevents substantial amounts of it from reaching the cell surface. Finally, we provide evidence that purified capsule can act as an external binding molecule that sequesters defensins to protect non-encapsulated bacilli from killing by HBD-3, indicating that both bacillus bound and free capsule can contribute to resistance from killing by cationic AMPs.

## Results

### Human alpha defensins have reduced bactericidal activity against an encapsulated *B*. *anthracis* strain compared to an isogenic non-encapsulated strain

**Fig 1:**
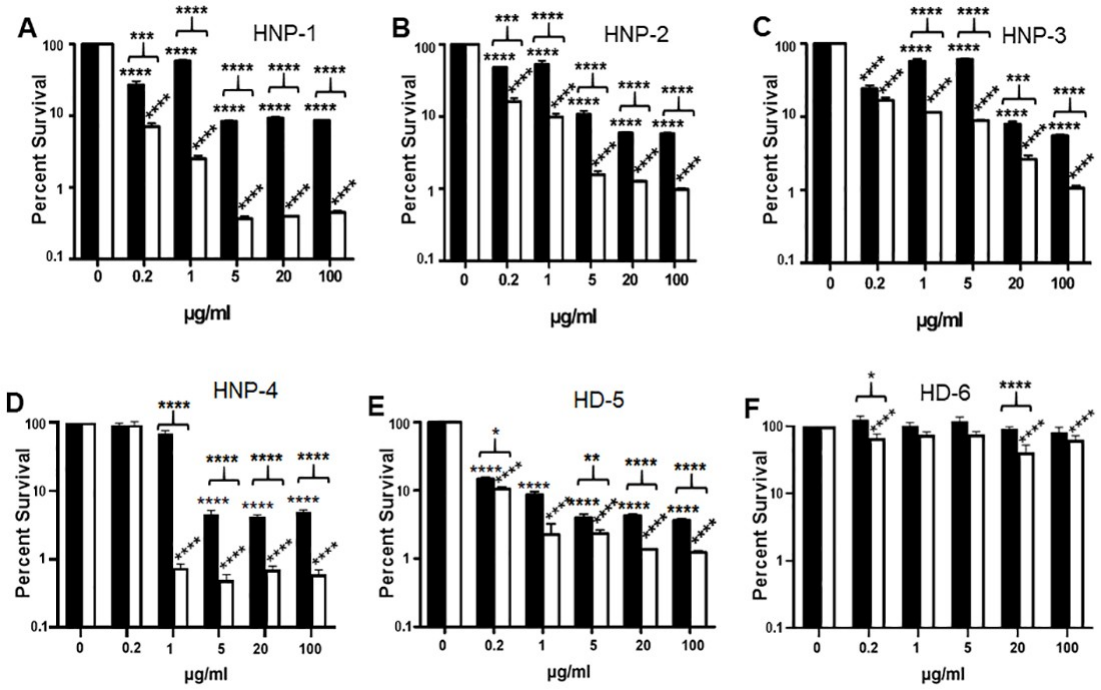
Human alpha defensins have reduced bactericidal activity against an encapsulated *B*. *anthracis* strain compared to an isogenic non-encapsulated strain. Encapsulated WT and non-encapsulated *capA* mutant bacilli were exposed to HNP-1 (A), HNP-2 (B), HNP-3 (C), HNP-4 (D), HD-5 (E), and HD-6 (F), at varying concentrations in triplicate tubes, incubated at 37°C in 5% CO_2_ for 2 h, and plated for CFU. Control tubes without defensins were incubated and plated in parallel. Survival percentages were determined by calculating the ratio of CFU with/CFU without defensin. Black bars represent WT and white bars represent *capA* mutant. Three experiments were run (n = 3). Results expressed as the mean + SEM from a representative experiment are shown. Significance of killing was determined by ANOVA with Tukey’s post-hoc test. Significance of differences in survival between the WT and *capA* strains was determined by two-tailed Student’s t-test (*p<0.05, **p<0.01, ***p<0.001, ****p<0.0001).

Alpha defensins expressed by human neutrophils have been reported to kill *B*. *anthracis* [40]. We assessed the antibacterial effects of the human alpha defensins individually on encapsulated WT and non-encapsulated *capA* mutant *B*. *anthracis* to see if encapsulation would prove protective. Bacilli from both strains were incubated with 0.2, 1, 5, 20, or 100 μg/ml HNP-1, HNP-2, HNP-3, HNP-4, HD-5, or HD-6 in triplicate tubes at 37°C with 5% CO_2_ for 2 h and then plated for CFU. Control tubes without defensins were incubated and plated in parallel. Survival percentages were calculated as the ratio of CFU with defensin/CFU without defensin. Mean data from a representative experiment are presented in Fig 1 (n = 3 experiments). HNP-1, HNP-2, HNP-3, HNP-4, and HD-5 were bactericidal against both strains at nearly all concentrations tested (Fig 1A–1E) while HD-6 had only minimal activity even at 100 μg/ml (Fig 1F). Bactericidal activity against the encapsulated WT strain was observed for HNP-1, HNP-2, HNP-3, and HD-5 at all concentrations tested (*p*< 0.0001) and for HNP-4 at 5, 20, and 100 μg/ml (*p*< 0.0001). HD-6 had no effect on the encapsulated WT strain. Bactericidal activity was observed against the non-encapsulated *capA* mutant strain at all concentrations for HNP-1, HNP-2, HNP-3, and HD-5 (*p*<0.0001). HNP-4 had bactericidal activity against the non-encapsulated *capA* mutant strain at all concentrations (*p*<0.0001) except 0.2 μg/ml, and HD-6 was bactericidal at 0.2, 20, and 100 μg/ml (*p*<0.0001). There was greater killing of the non-encapsulated *capA* mutant strain compared to the encapsulated WT strain with most of the alpha defensins. Greater killing of the non-encapsulated *capA* mutant was observed for HNP-1 and HNP-2 at all concentrations tested (*p*<0.0001 for 1–100 μg/ml and *p*<0.001 for 0.2 μg/ml, Fig 1A and 1B); for HNP-3 at 1–100 μg/ml (*p*<0.0001 for 1, 5 and 100 μg/ml and *p*<0.001 for 20 μg/ml, Fig 1C); for HNP-4 at 1–100 μg/ml (*p*<0.0001, Fig 1D); for HD-5 at 0.2–100 μg/ml (*p*<0.0001 for 20 and 100 μg/ml, *p*<0.01 for 5 μg/ml, and *p*<0.05 for 0.2 and 1 μg/ml, Fig 1E); and for HD-6 at 0.2, and 20 μg/ml (*p*<0.05 and *p*<0.0001, respectively, Fig 1F). These data indicate that capsule can protect *B*. *anthracis* from the bactericidal effects of human alpha defensins.

### Human beta defensins have reduced bactericidal activity against an encapsulated *B*. *anthracis* strain compared to an isogenic non-encapsulated strain

**Fig 2:**
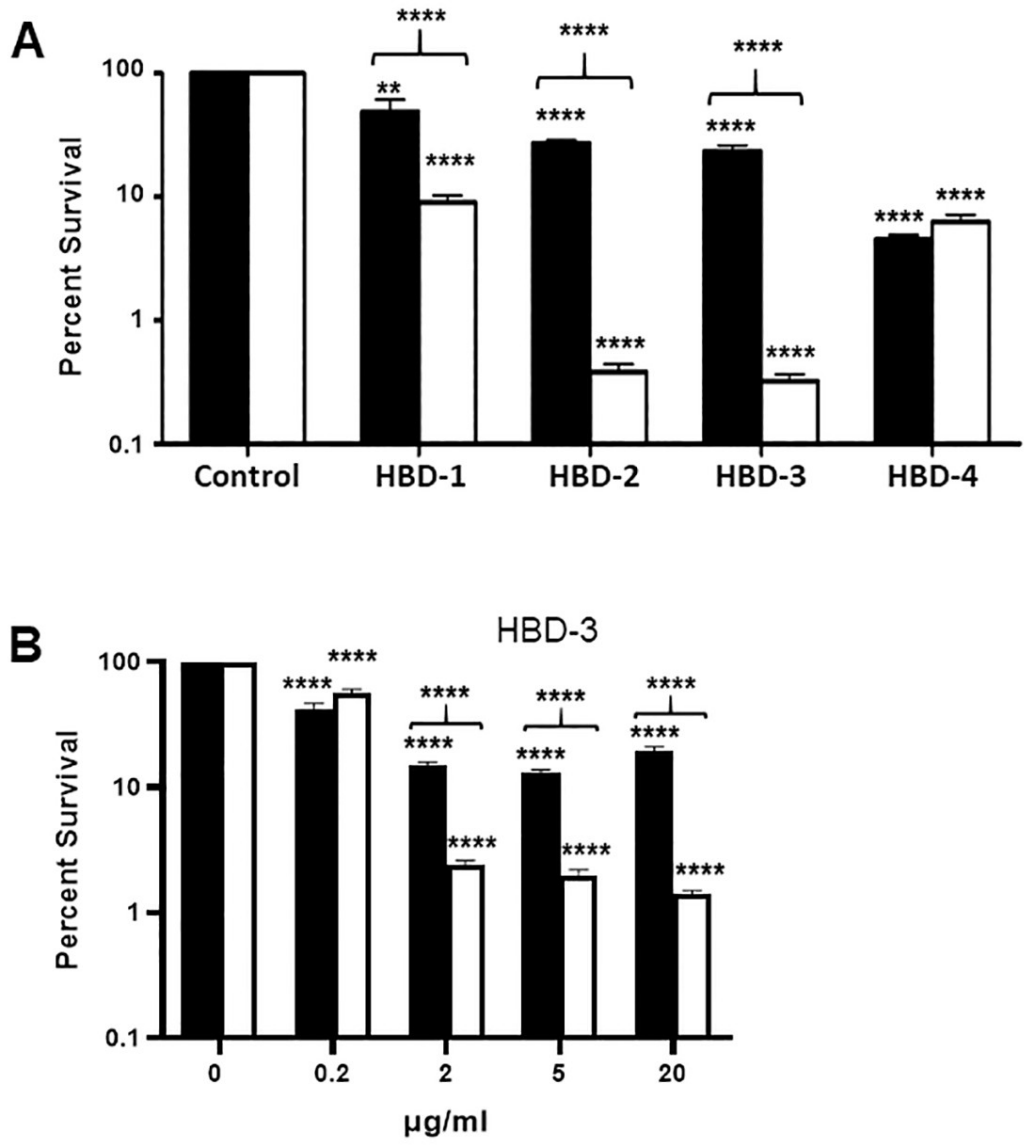
Human beta defensins have reduced bactericidal activity against an encapsulated *B*. *anthracis* strain compared to a non-encapsulated strain. Encapsulated WT and non-encapsulated *capA* mutant bacilli were incubated with human beta defensins 1, 2, 3, 4 (HBD-1, 2, 3, 4) at a final concentration of 20 μg/ml (A) or with varying concentrations of HBD-3 (B) in triplicate tubes at 37°C in 5% CO_2_ for 2 h and plated for CFU. Control tubes without defensins were incubated and plated in parallel. Survival percentages were determined by calculating the ratio of CFU with/CFU without defensin. Three experiments were run (n = 3). Results expressed as the mean + SEM from a representative experiment are shown. Black bars represent WT and white bars represent *capA* mutant. Significance of killing was determined by ANOVA with Tukey’s post-hoc test. Significance of differences in survival between the WT and *capA* strains was determined by two-tailed Student’s t-test (**p<0.001, ****p<0.0001).

The opportunistic human pathogen *Staphylococcus epidermidis* is encapsulated with a mixed D, L isomer glutamic acid γ-linked polymer that is similar to *B*. *anthracis* capsule. Encapsulated *S*. *epidermidis* has been shown to be resistant to killing by HBD-3 compared to a non-encapsulated mutant strain [44]. This suggested that encapsulated *B*. *anthracis* would also be resistant to human beta defensins. To test this, we compared the susceptibility of the encapsulated WT strain and non-encapsulated *capA* mutant strain to the bactericidal activity of HBD-1-4 individually. Bacilli from both strains were incubated with 20 μg/ml HBD-1, HBD-2, HBD-3, or HBD-4 in triplicate tubes at 37°C with 5% CO_2_ for 2 h and then plated for CFU. Control tubes without defensins were incubated and plated in parallel. Survival percentages were calculated as the ratio of CFU with defensin/CFU without defensin. Mean data from a representative experiment are presented in Fig 2 (n = 3 experiments). HBD-1, HBD-2, HBD-3, and HBD-4 were bactericidal against the encapsulated WT strain, resulting in 41% (*p*<0.01), 65% (*p*<0.0001), 71% (*p*<0.0001), and 95% (*p*<0.0001) killing respectively (Fig 2A). Bactericidal activity against the non-encapsulated *capA* mutant strain was also observed with HBD-1, HBD-2, HBD-3, and HBD-4 with 90%, 99%, 99%, and 94% (*p*<0.0001 for all) killing respectively (Fig 2A). These results demonstrate a dramatically greater bactericidal activity against the non-encapsulated *capA* mutant compared to the encapsulated WT strain for HBD-1-3 (Fig 2A, *p*<0.0001 for all) although no increased killing was observed with HBD-4 (Fig 2A). These data indicate that capsule can protect *B*. *anthracis* from several of the human beta defensins.

We titrated the bactericidal activity of HBD-3 and observed significant killing (*p*<0.0001) of both encapsulated WT and non-encapsulated *capA* mutant bacilli at concentrations from 0.2 to 20 μg/ml (Fig 2B). As before, greater killing was observed with the non-encapsulated *capA* mutant compared to the encapsulated WT strain (*p*<0.0001 for 2, 5, and 20 μg/ml, Fig 2B). Surprisingly, at 0.2 μg/ml the non-encapsulated *capA* mutant strain was slightly more resistant than the encapsulated WT strain although killing was modest for both (43% for *capA* mutant and 58% for WT) and the difference was not significant.

### Non-human AMPs have reduced bactericidal activity against an encapsulated *B*. *anthracis* strain compared to an isogenic non-encapsulated strain

**Fig 3:**
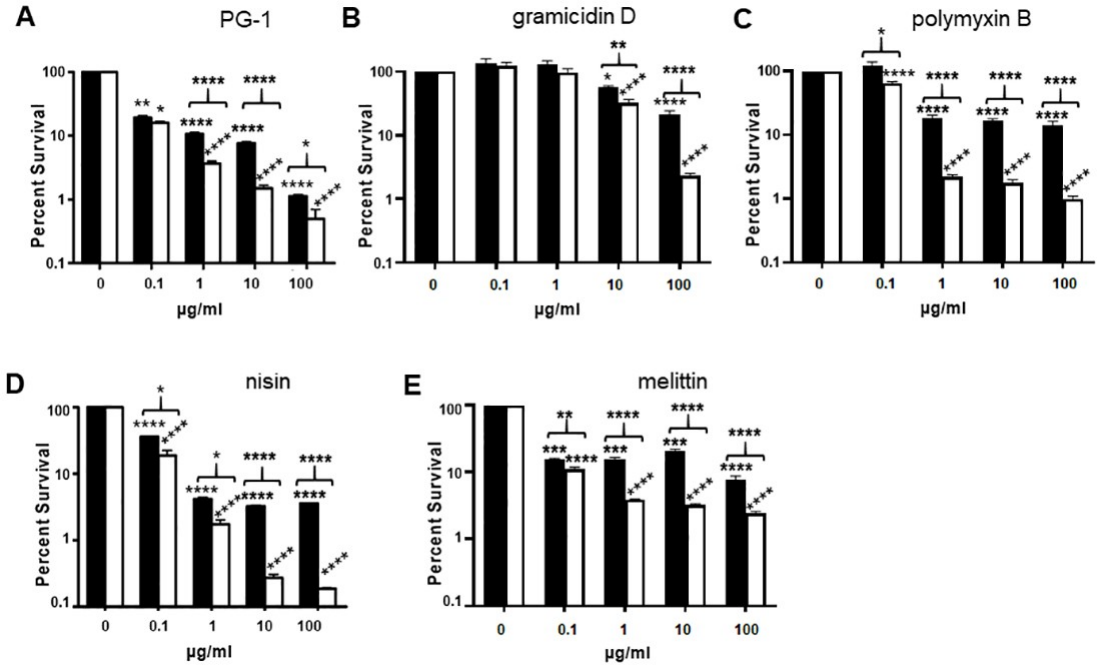
Various non-human cationic AMPs have reduced bactericidal activity against an encapsulated *B*. *anthracis* strain compared to a non-encapsulated strain. Encapsulated WT and non-encapsulated *capA* mutant bacilli were incubated with PG-1 (A), gramicidin D (B), polymyxin B (C), nisin (D), or melittin (E) in triplicate tubes at 0.1–100 μg/ml at 37°C in 5% CO_2_ for 2 h and plated for CFU. Survival percentages were determined by calculating the ratio of CFU with/CFU without AMP. Three experiments were run (n = 3). Results expressed as the mean + SEM from a representative experiment are shown. Black bars represent WT and white bars represent *capA* mutant. Significance of killing was determined by ANOVA with Tukey’s post-hoc test. Significance of differences in survival between the WT and *capA* strains was determined by two-tailed Student’s t-test (*p<0.05, **p<0.01, ***p<0.001, ****p<0.0001).

Since encapsulation afforded some protection from killing by several human defensins, we hypothesized that it might afford some protection from killing by AMPs from other species as well. To test this idea, porcine, bacterial, and insect AMPs were examined for their bactericidal activity against the encapsulated WT and non-encapsulated *capA* mutant *B*. *anthracis* strains. Bacilli from both strains were incubated with 0.1, 1, 10, or 100 μg/ml PG-1, gramidicidin D, polymyxin B, nisin, or melittin in triplicate tubes at 37°C with 5% CO_2_ for 2 h and then plated for CFU. Control tubes without AMPs were incubated and plated in parallel. Survival percentages were calculated as the ratio of CFU with AMPs/CFU without AMPs. Mean data from a representative experiment are presented in Fig 3 (n = 3 experiments). All AMPs tested were bactericidal against both the encapsulated WT and non-encapsulated *capA* mutant strains. PG-1 showed significant killing of encapsulated WT *B*. *anthracis* and the non-encapsulated *capA* mutant at 0.1–100 μg/ml (*p*<0.01 for WT and *p*<0.05 for *capA* at 0.1 μg/ml and *p*<0.0001 for both strains at 1–100 μg/ml, Fig 3A). The non-encapsulated *capA* mutant strain was more susceptible than the encapsulated WT strain at all concentrations tested, with significant differences at 1, 10 (*p*<0.0001), and 100 μg/ml (*p*<0.05). Gramicidin D was bactericidal for the encapsulated WT strain at 10 (*p*<0.05) and 100 μg/ml (*p*<0.0001) only (Fig 3B). Killing was also observed for the non-encapsulated *capA* mutant strain at 10 and 100 μg/ml gramicidin D (*p*<0.0001), but to a greater extent than for the encapsulated WT (*p*<0.01 for 10 μg/ml and *p*<0.0001 for 100 μg/ml, Fig 3B). Polymyxin B was bactericidal toward the encapsulated WT strain at 1–100 μg/ml (*p*<0.0001) and toward the non-encapsulated *capA* mutant strain at 0.1–100 μg/ml (*p*<0.0001). Greater killing of the non-encapsulated *capA* mutant was observed at 0.1–100 μg/ml (*p*<0.05 for 0.1 μg/ml, and *p*<0.0001 for 1–100 μg/ml, Fig 3C). Nisin was bactericidal toward both strains at 0.1–100 μg/ml (*p*<0.0001) with greater killing of the non-encapsulated *capA* mutant bacilli at all concentrations (*p*<0.05 for 0.1 and 1 μg/ml, and *p*<0.0001 for 10 and 100 μg/ml, Fig 3D). Melittin was bactericidal toward the encapsulated WT strain at 0.1–100 μg/ml (*p*<0.001 for 0.1–10 μg/ml, and *p*<0.0001 for 100 μg/ml). Melittin was also bactericidal toward the non-encapsulated *capA* mutant at 0.1–100 μg/ml (*p*<0.0001 for all). The non-encapsulated *capA* mutant strain was consistently more susceptible to killing by melittin than the encapsulated WT strain (*p*<0.01 for 0.1 μg/ml and *p*<0.0001 for 1–100 μg/ml, Fig 3E). Thus, as with many of the human alpha and beta defensins, encapsulation provided some protection from killing by AMPs from non-human species.

### Purified *B*. *anthracis* capsule binds human defensins

**Fig 4:**
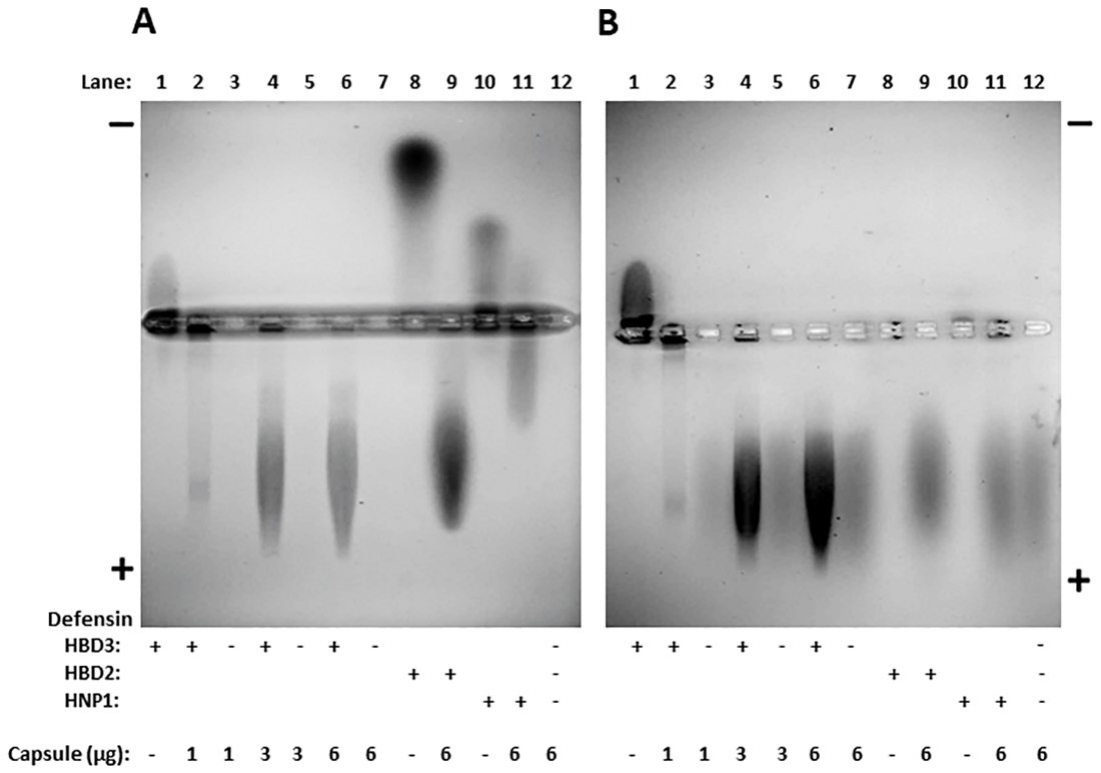
*B*. *anthracis* capsule binds human beta and alpha defensins. 3 μg of HBD-3, HBD-2, or HNP-1 was incubated with or without 1, 3, or 6 μg capsule for 30 min at 37°C. Samples were then electrophoresed in a 1% TAE agarose gel at 100 V at 4°C for 1 h. Defensins were stained with GelCode Blue (A) and then capsule was visualized in the same gel with Stains-All (B), as described in Materials and Methods. The white ring around the wells is an artifact due to the reflection of the overhead light off of the raised rims of the wells. Three experiments were run (n = 3). A representative gel is shown.

*B*. *anthracis* capsule is anionic and defensins are cationic and thus are likely to bind to each other. To determine if *B*. *anthracis* capsule binds human defensins, we performed an electrophoretic mobility shift assay with purified capsule and HBD-3, HBD-2, and HNP-1. Previous experiments have shown that capsule does not stain with GelCode Blue, but does stain with Stains-All [45]. In contrast, HBD-2, and HNP-1 stain with GelCode Blue, but not with Stains-All, while HBD-3 stains with both. Thus, sequential staining of the gel with GelCode Blue followed by Stains-All allowed us to visualize the positions of the defensins (Fig 4A) and the capsule (Fig 4B). As expected, the positively charged defensins migrated toward the anode (Fig 4A, lanes 1, 8, 10), while the negatively charged capsule migrated toward the cathode (Fig 4B, lanes 3, 5, 7, and 12). Incubation of HBD3, HBD-2, and HNP-1 with purified capsule prior to running on the gel caused a shift in their electrophoretic mobility towards the cathode, indicating that they bound to capsule (Fig 4A, compare lane 1 with lanes 2, 4, and 6, lane 8 with lane 9, and lane 10 with lane 11).

### The capsule layer surrounding encapsulated WT *B*. *anthracis* bacilli binds defensins

**Fig 5:**
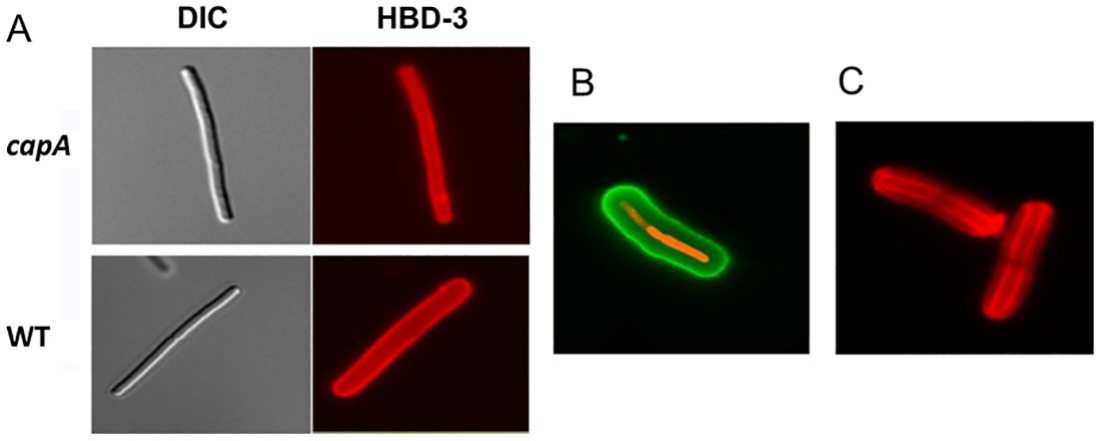
Detection of HBD-3 binding to *B*. *anthracis* bacilli. (A) Non-encapsulated *capA* mutant (upper quadrants) or encapsulated WT (lower quadrants) bacilli were incubated with 5 ug HBD-3. Left panels show differential interference contrast (DIC) microscopy images. Right panels show fluorescence microscopy images. HBD-3 binding was detected using rabbit anti-HBD-3 and AF594 conjugated goat anti-rabbit IgG antibody (red, magnification x 1,000). (B) Encapsulated WT bacilli were incubated with 5 ug Atto-594-labeled HBD-3 (red). Capsule was detected with FITC-labeled anti-capsule mAb (green). Magnification x 1,000. (C) Encapsulated WT bacilli were incubated with 20 ug Atto-594-labeled HBD-3. Magnification x 1,000.

Having demonstrated that purified capsule binds defensins *in vitro*, we hypothesized that the capsule layer surrounding encapsulated WT bacilli could also bind them. To investigate this, we added HBD-3 to non-encapsulated *capA* mutant bacilli and encapsulated WT bacilli and localized the HBD-3 using fluorescently labeled antibodies. We detected of HBD-3 on the cell wall of the non-encapsulated *capA* mutant bacilli, but only on the outer surface of the capsule of encapsulated WT bacilli (Fig 5A). However, this did not necessarily indicate that there was no HBD-3 on the cell wall of the WT bacilli because, IgG and IgM antibodies are unable to penetrate the capsule layer [46]. Therefore, we developed an alternative approach to localize HBD-3 interacting with encapsulated WT bacilli. We labeled HBD-3 with the red fluorescent dye Atto-594 prior to incubation with encapsulated WT bacilli. Fluorescence microscopy revealed that Atto-594-labeled HBD-3 was localized both on the cell wall of encapsulated WT bacilli (Fig 5B) and throughout the capsule layer (Fig 5C). Thus, HBD-3 can access and bind the cell wall of both strains of bacilli, which is consistent with its ability to kill both strains (Fig 2). However, in the case of the encapsulated WT bacilli, some of the HBD-3 is bound within the capsule layer, likely reducing the amount that reaches the cell wall.

### Encapsulation reduces the amount of defensin reaching the cell wall

**Fig 6:**
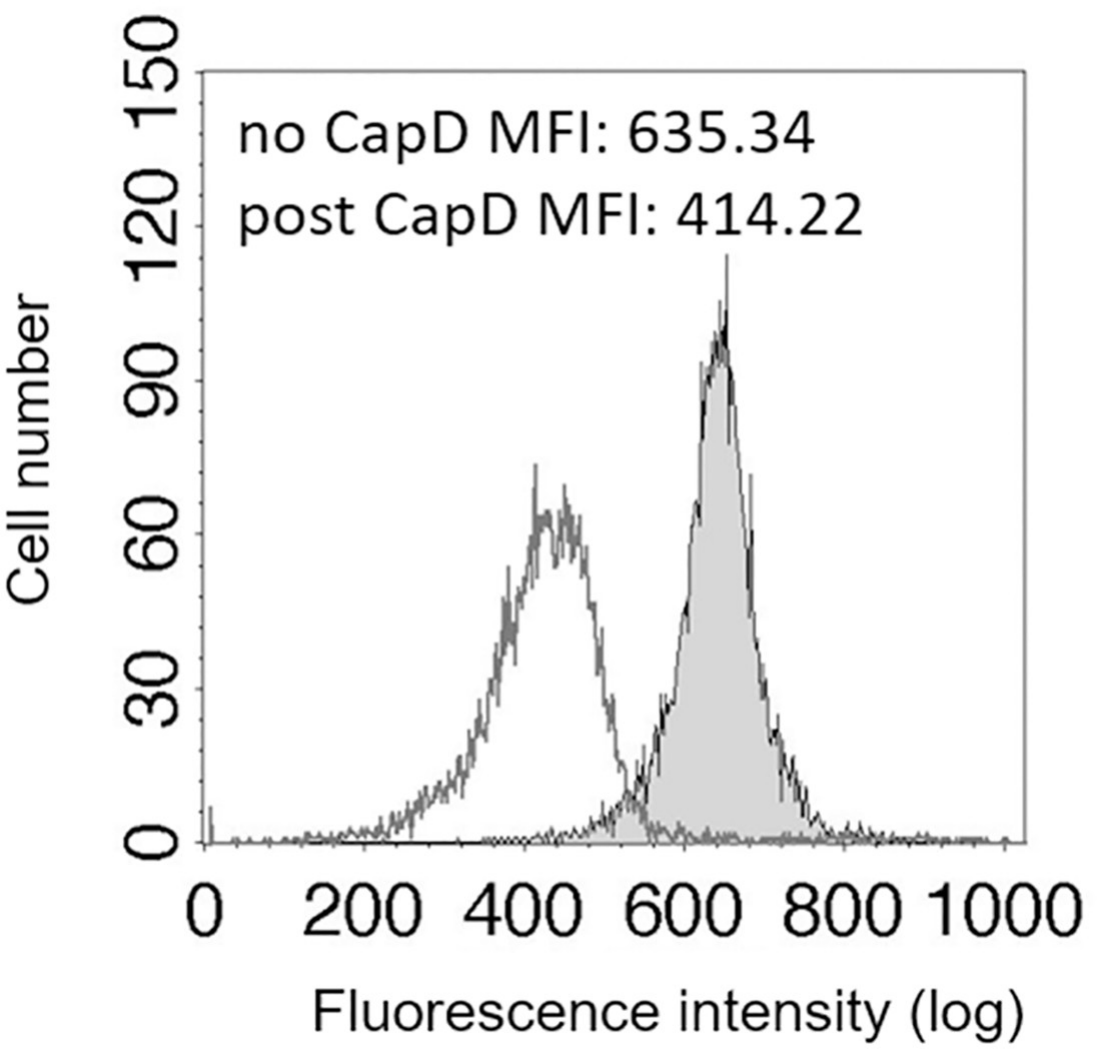
Encapsulation reduces the amount of HBD-3 reaching the bacillus cell wall. Encapsulated killed WT bacilli were incubated with 20 ug Atto-594-labeled HBD-3. A portion of the bacilli was subsequently treated with CapD to remove the capsule. Atto-594-labeled HBD-3 binding was measured by flow cytometry. The grey filled histogram represents bacilli without CapD treatment. The white filled histogram represents CapD treated bacilli. Four experiments were done (n = 4). Data from a single representative experiment are shown.

In order to demonstrate further that encapsulation reduces the amount of HBD-3 reaching the cell wall, we incubated Atto-594-labeled HBD-3 with encapsulated WT killed bacilli and then enzymatically removed the capsule from a portion of the bacilli by treatment with CapD. CapD treatment removes the capsule [11,47] and with it any Atto-594-labeled HBD-3 bound to it. After washing the bacilli, we measured remaining bound Atto-594-labeled HBD-3 by flow cytometry and compared it to that of the bacilli that weren’t treated with CapD. The mean fluorescence intensity was reduced on average by 32.7% ± 1.3% SEM after CapD treatment (*p* < 0.05, n = 4 experiments, Fig 6). These results indicate that capsule binding HBD-3 prevents a substantial portion of the HBD-3 from reaching the cell wall.

### Addition of purified capsule reduces bactericidal activity of human defensins against the non-encapsulated capA mutant strain

**Fig 7:**
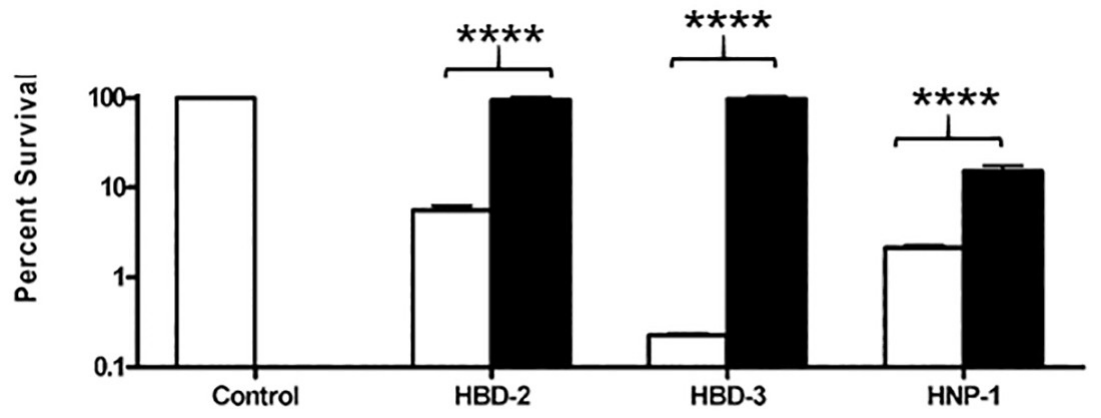
Exogenously added capsule reduces the bactericidal activity of human beta and alpha defensins against non-encapsulated *B*. *anthracis*. 20 μg/ml HBD-2, HBD-3, or HNP-1 was pre-incubated alone or with 1 mg/ml capsule for 30 min at 37°C before being added to non-encapsulated *capA* mutant bacilli in triplicate tubes. After incubation at 37°C in 5% CO_2_ for 2 h bacteria were plated for CFU. Control tubes without defensins were incubated and plated in parallel. Survival percentages were calculated by calculating the ratio of CFU with/CFU without defensin. Three experiments were run (n = 3). Results expressed as the mean + SEM from a representative experiment are shown. Black bars represent samples where capsule was pre-incubated with the defensin and the white bars represent defensins alone. Significance of differences in survival with and without capsule was determined by two-tailed Student’s t-test (****p<0.0001).

Since purified capsule can bind HBD-2, HBD-3, and HNP-1 (Fig 4) and the capsule layer of encapsulated WT bacilli can prevent substantial amounts of HBD-3 from reaching the cell wall (Fig 6), we hypothesized that purified free *B*. *anthracis* capsule could also inhibit the bactericidal activity of defensins by binding and sequestering them away from the bacillus cell wall. To test this idea, we pre-incubated 20 μg/ml HBD-2, HBD-3, and HNP-1 with or without 1mg/ml purified capsule for 30 min before adding them to non-encapsulated *capA* mutant bacilli. The bacilli were then incubated in triplicate tubes at 37°C with 5% CO_2_ for 2h and plated for CFU as before. Control tubes without defensins were incubated and plated in parallel. Survival percentages were calculated as the ratio of CFU with defensin/CFU without defensin. Mean data from a representative experiment are presented in Fig 7 (n = 3 experiments). Incubation of the non-encapsulated *capA* strain with 20 μg/ml HBD-2 alone resulted in 95% killing, but pre-incubation of HBD-2 with purified capsule completely eliminated its bactericidal activity with < 1% of the bacteria being killed (*p*<0.0001, Fig 7). Similarly, pre-incubation of HBD-3 with capsule decreased killing from >99% to < 1% (*p*<0.0001, Fig 7). Pre-incubation of HNP-1 with capsule reduced the killing of non-encapsulated *capA* mutant bacilli from 98% to 85% (*p*<0.0001), about the level of killing observed for encapsulated WT bacilli with HNP-1. These data suggest that free *B*. *anthracis* capsule as well as that bound to the surface of bacilli can reduce the bactericidal effect of defensins.

## Discussion

During infection *B*. *anthracis* bacilli become encapsulated and thereby resistant to phagocytic cell killing [9–11] and less stimulatory to dendritic cells [12]. Furthermore, there is evidence that capsule released by the bacilli during infection also contributes to pathogenesis [5,13]. In this study we sought to determine whether capsule, either surrounding the bacillus or free capsule released from the bacillus, contributes to virulence by inhibiting the bactericidal activity of defensins and other cationic AMPs. AMPs such as defensins are a critical first line of host defense. Bacteria have evolved many strategies for resisting AMPs including: electrostatic repulsion of cationic AMPs by increasing the net positive charge on the bacterial surface, degradation of AMPs, active removal of AMPs that enter cells via efflux pumps, and production of external AMP binding molecules that sequester them away from the bacterial membrane (reviewed in [42] and [43]. Previous work has provided evidence that *B*. *anthracis* employs two of these strategies: increasing the net positive charge on its surface and degradation of AMPs. The *dltABCD* operon in *B*. *anthracis* encodes a cell wall D-alanine esterification system that is responsible for alanylation of techoic acids, which makes the cell surface charge more positive. Inactivation of the *dltABCD* operon in the non-encapsulated Sterne strain resulted in increased susceptibility to HNP-1, HNP-2, HBD-2 and other cationic AMPs, and attenuation in a mouse model [38]. *B*. *anthracis* can also increase its surface charge by the production of lysylphosphatidylglycerols; blocking production of these phospholipids by inactivation of the *mprF* gene in the non-encapsulated ΔANR strain led to increased susceptibility to LL-37 and HNP-1 [48]. There is also evidence that *B*. *anthracis* can degrade human LL-37 [49]. Loss of the *clpX* gene in the non-encapsulated Sterne strain led to increased sensitivity to LL-37 and HNP-2, likely due to decreased expression of extracellular proteases [50]. In this report we provide evidence that *B*. *anthracis* employs a third AMP resistance strategy, production of an external sequestration molecule, i.e. capsule.

To see if encapsulation conferred resistance to human defensins, we assessed and compared the bactericidal effects of human alpha and beta defensins on a fully encapsulated WT strain and an isogenic non-encapsulated *capA* mutant strain. When we examined the human alpha defensins, HNP-1-4 and HD-5 showed significant antimicrobial activity against the encapsulated WT strain but were even more active against the non-encapsulated *capA* mutant strain. This contrasts with a report by Montville et al. showing no activity of HNP-1 and HNP-2 against the fully virulent encapsulated Pasteur and Vollum strains of *B*. *anthracis* they tested [39]. However, they did report that HNP-1 and HNP-2 had activity against the non-encapsulated Sterne strain [39], which supports our hypothesis that encapsulation is protective. Since they used agar diffusion assays to measure bactericidal activity, differences in assay methods may explain the discrepancy in results for the encapsulated strains. Work by Mayer-Scholl et al. indicated that human neutrophils kill *B*. *anthracis* via the alpha defensins present in their granules [40]. Mayer-Scholl et al. did not identify which alpha defensin was responsible, but we have shown that HNP-1-4 individually can kill *B*. *anthracis*. When we examined the effects of human beta defensins, we found that HBD-1-3 killed non-enapsulated *capA* mutant much more efficiently than the encapsulated WT strain. It was previously reported that HBD-3 could kill the non-encapsulated Sterne strain of *B*. *anthracis* while HBD-1 and HBD-2 could not [37]. In contrast, our results show that HBD-2 is as active as HBD-3 at 20 μg/ml against the non-encapsulated *capA* strain with >99% killing. The difference could be due to the different assays employed or possibly, but less likely, due to differences between the Sterne and *capA* mutant strains. Theta defensins produced by non-human primates that were humanized have been reported to have similar activity against an encapsulated and unencapsulated non-toxinogenic *B*. *anthracis* strain when tested in vitro [51]. However, in mouse experiments the theta defensins were only effective against a fully virulent encapsulated strain when given together with the spore inoculum and not when given after the infection with spores or against an infection in which mice were infected with encapsulated bacilli. This could be explained by the greater activity of the theta defensins against early germinated bacilli which are likely not fully encapsulated than against fully encapsulated bacilli.

We also examined the bactericidal activity of various non-human AMPs. PG-1, gramicidin D, polymyxin B, nisin, and melittin killed greater percentages of the non-encapsulated *capA* mutant than the encapsulated WT strain. The differences in killing were significant at most concentrations for all the AMPs tested except gramicidin D. Contrary to our results, Lisanby et al. determined that PG-1 killed encapsulated bacilli more effectively than non-encapsulated bacilli *in vitro* using an agar radial diffusion assay [52]. This may also be due to the difference in the assays used. Consistent with our results, Montville et al. found that the non-encapsulated Sterne strain was much more sensitive to nisin than the encapsulated Pasteur strain though surprisingly, the encapsulated Vollum strain was more sensitive than Sterne [39]. In this study agar diffusion assays were also used and so differences may be due to the different assays used. Interestingly, a *Klebsiella pneumoniae* mutant that doesn’t express anionic capsular polysaccharide (CPS) was more sensitive to killing by HNP-1, HBD-1, and polymyxin B than an isogenic strain expressing CPS [53]. Taken together, these results and ours suggest that encapsulation with anionic material may play a role in protection against the microbicidal activity of cationic AMPs.

Since capsule is anionic and the defensins are cationic, it was expected that they would bind to each other and that was indeed the case. In an electrophoretic mobility shift assay, HNP-1, HBD-2 and -3 migrated toward the anode in the absence of capsule and toward the cathode in its presence (Fig 4). Interestingly, the individual defensins exhibited differences in migration toward the anode reflecting differences in charge and size. HBD-2 migrated furthest due to its higher positive charge while HNP-1 migrated more slowly due to its lower positive charge. HBD-3 exhibited minimal migration towards the anode. This may be because HBD-3 can dimerize [54], which could slow its migration.

We demonstrated HBD-3 binding to the cell wall of the non-encapsulated *capA* mutant strain by fluorescence microscopy using an anti-HBD3 antibody. Determining where HBD-3 bound on encapsulated WT bacilli was less straightforward because IgG and IgM cannot penetrate the capsule layer [46]. By using fluorescently labeled HBD-3 we were able to show that HBD-3 bound not only to the outer surface of the capsule layer, but within the capsule layer, and on the cell surface. When we incubated fluorescently labeled HBD-3 with encapsulated WT bacilli and then removed the capsule using CapD, we were able to demonstrate by flow cytometry that a substantial amount of HBD-3 is bound up in the capsule. Thus, a substantial amount of HBD-3 never reaches the cell surface of encapsulated WT bacilli, a result consistent with the WT strain’s relative resistance to HBD-3. These results demonstrate sequestration of HBD-3 by the capsule surrounding encapsulated WT bacilli.

*B*. *anthracis* sheds large amounts of capsule during infection and shed capsule has been reported to accumulate to >0.5 mg/ml in the blood of mice [55] and up to 1 mg/ml in the blood of rhesus macaques [56]. Furthermore, released capsule has been shown to contribute to virulence in a mouse model [5]. We thought it possible that shed capsule could act as an external sequestration molecule for cationic AMPs. We were able to rescue non-encapsulated *capA* mutant bacilli from killing by HBD-2 and HBD-3 completely by adding purified capsule to the defensins (Fig 7). While adding purified capsule to HNP-1 did not rescue non-encapsulated *capA* mutant bacilli, it did reduce the level of killing to about that of encapsulated WT bacilli treated with HNP-1. These data support the idea that free capsule binds defensins, effectively sequestering them away from the bacilli and inhibiting their activity. The varying levels of inhibition by free capsule may be due to inherent properties of the individual defensins, such as their charge and isoelectric points (pI). Capsule’s pI is 2.0 whereas HNP-1’s is 8.37 and HBD-2 and HBD-3’s are 9.19 and 10.63, respectively. It is possible that the higher pIs of HBD-2 and HBD-3 cause them to bind more efficiently to capsule than HNP-1 does. In an interesting parallel, purified anionic CPSs from *K*. *pneumoniae*, *Streptococcus pneumoniae*, and *Pseudomomas aeruginosa* have been shown to reduce the sensitivity of a non-encapsulated *K*. *pneumoniae* mutant strain to polymyxin B and HNP-1 [57]. Taken together, our results indicate that both capsule attached to bacilli and free capsule can sequester cationic AMPs, thereby inhibiting their activity and conferring protection.

Other *Bacillus* species, other bacteria, archea, and some eukaryotes produce γ-linked mixed D, L glutamic acid polymers that are similar to *B*. *anthracis* capsule [58]. Typically these organisms secrete the polymers into their environment to sequester heavy metals or decrease local salt concentrations to make the environment more favorable. Our results suggest that capsule shed by *B*. *anthracis* may serve a similar purpose during infection, sequestering defensins and other cationic AMPs to make the host a more favorable environment and contribute to virulence. Interestingly, two related species, *B*. *subtilis* and *B*. *licheniformis* (formerly designated *B*. *subtilis* ATCC 9945a), which produce secreted γ-linked D, L glutamic acid polymers, are not pathogenic in humans except under very rare circumstances [59]. This is likely because, in addition to these strains lacking the anthrax toxins, the *B*. *subtilis* and *B*. *licheniformis* polymers are far more stimulatory to human immune cells than *B*. *anthracis* capsule and are also much more readily degraded by human proteases [60]. *B*. *anthracis* capsule’s greater protease resistance allows it to accumulate in host tissues and possibly sequester cationic AMPs.

*B*. *anthracis* is a highly virulent pathogen with multiple strategies for resisting the host immune system. In this study, we demonstrate a novel means of resistance provided by the capsule, sequestration of host AMPs such as defensins to reduce their bactericidal activity. Defensins serve other immune functions in addition to their antimicrobial activity [61], including acting as chemoattractants for immune cells [18,19,62]. It will be interesting to see if sequestration by capsule interferes with this defensin function as well. It is already clear that capsule interferes with the host immune response in multiple ways, allowing encapsulated bacilli to resist phagocytosis, delay maturation of dendritic cells, and, as our current study shows, resist killing by many cationic AMPs. Together, these results expand our knowledge of the ways that capsule contributes to pathogenesis and highlight the need for countermeasures focusing on this critical virulence factor.

## Materials and methods

### Bacterial strains and growth conditions

*B*. *anthracis* Ames and the isogenic capsule deficient *capA* mutant strain [13] were from the USAMRIID collection. Bacilli were prepared by inoculating brain heart infusion (BHI) broth containing 0.8% sodium bicarbonate with 1–5 x 10^7^ spores/ml and incubating at 37°C with 5% CO_2_ for 90 min on a reciprocal shaker at 150 rpm. This resulted in >99% germination as measured by heat-sensitivity. Bacilli were centrifuged at 14,300 x g, washed with PBS and resuspended in 10 mM sodium phosphate buffer, pH 7.4. The presence or absence of encapsulation was verified by India ink staining. Before use, spores were activated by heat shock at 65°C for 30 min. For the flow cytometry experiments, bacilli were inactivated by treatment with 4% paraformaldehyde for 3 h.

### AMP microbicidal activity assays

Bactericidal activity assays were done as previously described [63]. Briefly, 5 x 10^6^ bacilli /ml were suspended in cold 10 mM sodium phosphate buffer. Assays were performed in triplicate in 1 ml Eppendorf tubes. Each tube contained 35 μl of assay medium (6.9 ml of 10 mM sodium phosphate buffer and 0.1 ml of trypticase soy broth) pre-warmed to 37°C, 10 μl of bacteria resulting in a final concentration of 1 x 10^6^ bacilli/ml, and 5 μl of a 10x stock of AMP except for the control tubes, which received no AMP. The tubes were mixed gently and incubated at 37°C under 5% CO_2_ for 2 h at which time the reaction was stopped by adding 450 μl of ice-cold 0.15 M sodium chloride. Viable colony forming units (CFU) were determined by serial dilution and plating on tryptic soy agar plates. Results shown in Figs 1, 2, 3 and 7 are for representative experiments (n = 3 experiments). In each experiment, triplicate samples were run. CFU on duplicate plates were counted and averaged to determine average CFU/sample. The ratio of the average CFU in the AMP test sample to that in the control tube was used to calculate the percentage survival for each of the triplicate samples. These percentages were averaged to calculate the mean percentage and SEM for the experiment. Significant results were obtained in three separate experiments for each AMP. Mean survival percentages, SEM, and *p* values from representative experiments are shown in the figures. Polymyxin B, gramicidin D, nisin, and melittin were obtained from Sigma-Aldrich (St. Louis, MO). Protegrin (PG-1) and recombinant HBD-1-3 were obtained from Peprotech (Rocky Hill, NJ) and recombinant HBD-4 was obtained from Peptides International (Louisville, KY). HNP-1-4, and HD-5-6 were synthesized as previously described [64,65].

The effect of purified free capsule on bactericidal activity was determined by preincubating *B*. *anthracis* capsule with defensins for 30 min at 37°C before addition to the assay buffer and bacteria as described above for a final concentration of 1 mg/ml of capsule and 20 μg/ml of defensin. After incubation for 2 h at 37°C in 5% CO_2_ the reaction was stopped and bacterial viability determined as described above. Capsule was purified from *B*. *anthracis* Ames bacilli as previously described [45].

### Electrophoretic mobility shift assay

3 μg of defensin were incubated alone or with 1, 3, or 6 μg *B*. *anthracis* capsule in 10 mM sodium phosphate buffer in a final volume of 10 μl. The mixtures were incubated at 37°C for 30 min, followed by addition of 1.1 μl of BlueJuice gel loading buffer (Invitrogen, Carlsbad, CA). Samples were then electrophoresed in a 1% agarose gel with Tris-acetate-EDTA buffer (Mobio Laboratories Inc., Carlsbad, CA) at 100 V at 4°C with pre-refrigerated running buffer for 1h. The gel was washed in water for 5 min and fixed with 10% ethanol, 3% acetic acid for 15 min. Proteins in the gel were stained with GelCode Blue (Pierce Biotechnology, Inc., Rockford, IL) for 2 h and destained in water for 1 h. The gel was scanned, washed in 10% propanol, 10% formamide for 15 min, and stained for capsule overnight with 0.02% Stains-All (Sigma-Aldrich) in 10% propanol, 10% formamide. The gel was destained in the dark with eight changes of 10% propanol, 10% formamide over 2 days before rescanning.

The isoelectric point of capsule and the defensins was calculated using a sequence manipulation suite http://bioinformatics.org/sms2/protein_iep.html.

### Microscopy and flow cytometry

*B*. *anthracis* Ames and *capA* mutant bacilli were grown separately in BHI containing 0.8% sodium bicarbonate and 5% CO_2_ for 3 h at 37°C. Bacilli were then washed with 10 mM sodium phosphate buffer and incubated with HBD-3 in 10 mM sodium phosphate buffer at a concentration of 5 or 20 μg/ml for 1h at 37°C. Bacilli were then washed twice by centrifugation at 12,000 x g and resuspended in PBS. HBD-3 bound to bacilli was detected by incubation with rabbit anti-HBD3 (1:1,000, Peprotech) for 1h at room temperature followed by incubation with AF594-conjugated goat anti-rabbit antibody (1:1,000 ThermoFisher Scientific, Waltham, MA, cat# A-11012) for 30 min at room temperature. In some experiments, HBD-3 was fluorescently labeled using a Lightning-Link Atto-594 Conjugation Kit (Novus Biologicals, Littleton, CO) per the manufacturer’s instructions before addition to the bacilli. Atto-594-labeled HBD-3 binding to bacilli was detected by fluorescence microscopy using a TRITC filter set. The capsule surrounding WT bacilli was detected with a FITC conjugated anti-capsule monoclonal antibody (FDF-1B9-FITC) as previously described [66]. Differential interference contrast (DIC) and fluorescent images were captured with an Eclipse TE2000 microscope (Nikon, Columbia MD), equipped with a Spot RT digital camera (Diagnostic Instruments, Inc., Sterling Heights, MI) and images were processed with QED In-Vivo Software (Media Cybernetics, Silver Spring, MD). In order to determine how much Atto-594-labeled HBD-3 bound to the capsule, encapsulated bacilli were incubated with CapD to remove the capsule as previously described [11]. Briefly, 1 x 10^8^ encapsulated bacilli in 1 ml were incubated with 20 μg Atto-594-labeled HBD-3 in Dulbecco’s Modified Eagle Medium (DMEM, ThermoFisher Scientific) for 30 minutes, washed by centrifugation, and resuspended in DMEM. An aliquot was then treated with 50 μg/ml CapD and incubated for 20 min at 37°C. Controls were incubated with DMEM alone. Treated and control bacilli were washed by centrifugation and resuspended in PBS for flow cytometry. Flow cytometry was performed with a FACSCalibur (BD Biosciences, Billerica, MA) and the data was analyzed using Cell Quest Pro software (BD Biosciences).

### Statistics

Differences between AMP treatment and untreated control groups for each strain were compared using analysis of variance (ANOVA) with Tukey’s post-hoc test using SAS software (SAS Institute Inc., Cary, NC). For all comparisons, a test for equality of variances was conducted. For those comparisons that failed the equality of variances test (*p* ≤ 0.05), a Satterthwaite estimate for unequal variances was used to determine the decision rule regarding the comparison of means via the t-test, and the associated *p* value was presented. Differences between strains at each concentration of AMP and between preincubation with and without capsule were compared using a two-tailed t test (GraphPad Software, La Jolla, CA). The difference in average mean fluorescence intensity with and without CapD treatment was compared using a two-tailed t test (GraphPad Software).

## Supporting information

S1 Data Excel spreadsheet containing, in separate sheets, the underlying numerical data and statistical analysis for Figure panels 1A, 1B, 1C, 1D, 1E, 1F, 2A, 2B, 3A, 3B, 3C, 3D, 3E, 6, and 7. (XLSX)
